# Risk factors for delayed perineal wound healing and its impact on prolonged hospital stay after abdominoperineal resection

**DOI:** 10.1186/s12957-019-1768-4

**Published:** 2019-12-21

**Authors:** Chu-Cheng Chang, Yuan-Tzu Lan, Jeng-Kai Jiang, Shih-Ching Chang, Shung-Haur Yang, Chun-Chi Lin, Hung-Hsin Lin, Huann-Sheng Wang, Wei-Shone Chen, Tzu-Chen Lin, Jen-Kou Lin

**Affiliations:** 1 Division of Colon & Rectal Surgery, Department of Surgery, Taipei Veterans General Hospital, No. 201, Section 2, Shih-Pai Road, Beitou District, Taipei, 11217 Taiwan; 2 Department of Surgery, National Yang-Ming University, Taipei, Taiwan; 3 En Chu Kong Hospital, Taipei, Taiwan

**Keywords:** Abdominoperineal resection, Perineal wound, Primary closure, Hypoalbuminemia, Hospital stay

## Abstract

**Background:**

Perineal wound complications are a long-lasting issue for abdominoperineal resection (APR) patients. Complication rates as high as 60% have been reported, with the most common complication being delayed perineal wound healing. The aim of this study was to identify risk factors for delayed perineal wound healing and its impact on prolonged hospital stay.

**Methods:**

We included low rectal tumor patients who underwent APR at a referral medical center from April 2002 to December 2017; a total of 229 patients were included. The basic characteristics and surgical outcomes of the patients were analyzed to identify risk factors for delayed perineal wound healing (> 30 days after APR) and prolonged hospital stay (post-APR hospital stay > 14 days).

**Results:**

All patients received primary closure for their perineal wound. The majority of patients were diagnosed with adenocarcinoma (*N* = 213, 93.1%). In the univariate analysis, patients with hypoalbuminemia (albumin < 3.5 g/dL) had an increased risk of delayed wound healing (39.5% vs. 60.5%, *P* = 0.001), which was an independent risk factor in the multivariable analysis (OR 2.962, 95% CI 1.437–6.102, *P* = 0.003). Patients with delayed wound healing also had a significantly increased risk of prolonged hospital stay (OR 6.404, 95% CI 3.508–11.694, *P* < 0.001).

**Conclusions:**

Hypoalbuminemia was an independent risk factor for delayed wound healing, which consequently led to a prolonged hospital stay. Further clinical trials are needed to reduce the incidence of delayed perineal wound healing by correcting albumin levels or nutritional status before APR.

## Background

Since Miles’ first description of abdominoperineal resection (APR) surgery in 1908 [1], APR has become the standard surgical treatment for low rectal and anal cancer, especially when sphincter-sparing surgery is not an option. Although the removal of the rectum, anal canal, perineum skin, and sometimes nearby organs decreases the incidence of circumferential margin involvement, removal is also associated with a 29–64% perineal wound complication rate after massive removal of a pelvic organ [2–5]. Large amounts of empty space in the pelvic cavity following large-scale destruction of the pelvic floor, high bacterial counts in the perineal area, and the closure of the perineal wounds with tension were considered factors contributing to complications [6, 7]. Risk factors associated with increased perineal wound complications include patient-related factors such as diabetes [8], high body mass index (BMI) [8], hypoalbuminemia [4], and surgery-related factors such as extralevator APR [3], intraoperative perforation [3], and supine position during the perineal phase [9]. For locally advanced anorectal cancer, a large tumor size and the application of preoperative radiotherapy also increase the risk of a perineal wound complication [2, 4, 8]. To reduce the perineal wound complication rate, flap reconstruction was introduced as an alternative procedure for closure of the perineal wound [10, 11], but it is generally only utilized in patients receiving an extended perineal excision or neoadjuvant radiotherapy. In general practice, primary closure is used in the majority of perineal wound cases [12, 13].

Perineal wound complications include superficial or deep infection, abscess formation, wound disruption, hematoma formation, or persistent sinus discharge. Once a complication occurs, it can result in additional costs and a prolonged hospital stay, especially when delayed wound healing follows these complications. Delayed perineal wound healing is the most common perineal wound complication [2, 4], and various risk factors have been demonstrated to be related to delayed perineal wound healing. Patient-related factors such as hypoalbuminemia [4], a history of smoking [4] and alcohol consumption [14] were found to increase the risk of delayed perineal wound healing. Treatment-related factors such as a longer operation time [15], intraoperative hypothermia [15], and preoperative radiotherapy [2, 4, 12, 14] have also been regarded as contributing factors. The incidence of delayed perineal wound healing is as high as 24–51% [2, 4, 12, 15]; however, the time period to define delayed wound healing differs among previous studies, ranging from 6 weeks to 3 months after primary surgery [2, 4, 12, 14, 15]. Thus, a direct comparison among these studies is difficult.

The aim of this study is to review the outcomes of patients receiving APR with primary closure of the perineal wound at a single institution and identify risk factors for delayed perineal wound healing and its association with prolonged hospital stay.

## Patients and methods

The study protocol was reviewed and approved by the Institute of Research Board of Taipei Veterans General Hospital (Institutional Review Board (IRB) no. 2018-09-003BC). All clinical data of the patients who underwent APR between April 1, 2002, and December 31, 2017, at Taipei Veteran General Hospital were collected retrospectively. We included patients receiving conventional APR with primary closure of the perineal wound. No extralevator APR cases were included. All APR procedures were performed by colorectal surgeons with more than 5 years of clinical experiences in their field of specialization.

Data were collected from inpatient medical records and outpatient clinical records. The following patient characteristics were analyzed: age at diagnosis, gender, BMI (defined > 25 kg/m^2^ as overweight and < 18.5 kg/m^2^ as underweight according to World Health Organization (WHO) classification), smoking history (current smokers and previous smokers), and comorbidities (such as hypertension, congestive heart failure, uremia, liver cirrhosis, and chronic obstructive pulmonary disease). The preoperative albumin (< 3.5 g/dL considered hypoalbuminemia) and carcinoembryonic antigen (CEA) (> 5 ng/ml considered elevated) levels of patients were recorded. The following intraoperative parameters were also recorded: operation time, estimated blood loss, method of operation (laparoscopic surgery or traditional laparotomy), and concomitant resection of other pelvic organs. Histopathology of the tumor and application of neoadjuvant radiotherapy were also retrieved from medical records. Neoadjuvant radiotherapy was administered using a long-course radiotherapy in patients with adenocarcinoma with a total dose of 4500 cGy over 4 weeks [16]. Definitive surgical resection was performed at 6 to 8 weeks after the completion of the neoadjuvant radiotherapy.

During APR, the abdominal and perineal phases were both performed with the patient in the supine lithotomy position. Abdominal phase was performed first following the principle of total mesorectal excision. Then, the operation moved to the perineal phase. After removal of the specimen through the perineum, a drainage tube was routinely inserted into the presacral space through the perineum. Primary closure of the perineal wound was performed in layers, and the abdomen was closed with the stoma matured. For laparoscopic APR, the abdominal phase was performed using laparoscopy, but the perineal phase was performed with the same procedure as the traditional open APR.

The primary end point was failure of the perineal wound to heal 30 days after the operation. The secondary end point was prolonged postoperative hospital stay (more than 14 days after the operation). All perineal wound-related complications, including infection, dehiscence, deep and pelvic abscess, bleeding, and persistent sinus drainage, during follow-up were also recorded.

Patients were followed-up with at the outpatient department at 2-week intervals until complete healing of the perineal wound. Then, follow-ups were scheduled for 3-month intervals for the first 2 years, followed by 6-month intervals until 5 years after the operation.

### Statistical analyses

Univariate analysis of perineal wound complications and categorical variables was performed using the chi-squared test or Fisher’s exact test. The continuous variables were analyzed with Student’s *t* test. The median value of blood loss and operation time was defined as the cutoff value in the analysis and compared using a nonparametric test. All predictors identified in the univariate analysis with a *p* value less than 0.10 were candidate variables for inclusion in the multivariable model to identify independent predictors of delayed perineal wound healing and prolonged hospital stay. A *p* value of less than 0.05 was considered statistically significant. All analyses were performed with IBM SPSS Statistics, version 24 (IBM Corp., Armonk. NY, USA).

## Results

A total of 229 patients were enrolled; Table 1 lists the clinicopathological characteristics of the patients. The median age of all enrolled patients was 69.5 ± 13.0 (range 19–95) years, and 146 (63.8%) were male. The most common indication for APR was adenocarcinoma of the anorectum, which was identified in 213 (93.1%) patients. Approximately one third of our patient group received neoadjuvant radiotherapy before surgery. Among the 86 overweight patients, six were obese with a BMI > 30 kg/m^2^. Sixty-one patients (26.6%) had a history of smoking, including 38 (16.6%) active smokers and 23 (10%) previous smokers. The most common comorbidity was hypertension, which was present in 99 (43.2%) patients, followed by diabetes in 39 (17.0%) patients and congestive heart failure in 37 (16.2%) patients.

**Table 1:** Clinicopathological characteristics of 229 patients receiving abdominoperineal resection

	No. (%)
Age (years old)	
Median ± S.D.	69.6 ± 13.0
Range	19–95
Gender	
Male	146 (63.8)
Female	83 (36.2)
Smoking	
Current smoker	38 (16.6)
Previous smoker	23 (10.0)
BMI (kg/m^2^)	
Median ± S.D.	23.9 ± 3.84
Range	15–41
Underweight (< 18.5)	9 (3.9)
Overweight (> 25)	86 (37.6)
Comorbidities	
Hypertension	99 (43.2)
Diabetics	39 (17.0)
Congestive heart failure	37 (16.2)
COPD	10 (4.4)
Uremia	9 (3.9)
Liver cirrhosis	3 (1.3)
Hypoalbuminemia (< 3.5 g/dL)	38 (16.6)
Elevated CEA level (> 5 ng/mL)	79 (34.5)
Neoadjuvant radiotherapy	76 (33.2)
Pathology of anorectum	
Adenocarcinoma	213 (93.1)
SCC	6 (2.6)
Melanoma	6 (2.6)
GIST	3 (1.3)
Neuroendocrine carcinoma	1 (0.4)

*BMI* body mass index, *COPD* chronic obstructive pulmonary disease, *CEA* carcinoembryonic antigen, *SCC* squamous cell carcinoma, *GIST* gastrointestinal stromal tumor, CCRT concurrent chemoradiotherapy

As shown in Table 2, the median operation time was 240 min (range 115–590 min), and the median estimated blood loss was 250 ml (range 30–2300 ml). A laparoscopic operation was performed on 28 (12.2%) patients. Thirty-two patients (14.0%) underwent resection of adjacent pelvic organs during APR. Among these patients, 21 were female who underwent a partial vaginectomy, and 11 were male who underwent a partial prostatectomy or resection of the seminal vesicle. Perineal wound-related complications occurred in 91 (39.7%) patients, with 149 events. Delayed wound healing (> 30 days) was the most common perineal wound-related complication, occurring in 86 patients (37.6%). Other perineal wound-related complications included 27 (11.8%) superficial wound infections, 22 (9.6%) wound dehiscence events, 7 (3.1%) deep or pelvic abscesses, 3 (1.3%) persistent discharging sinuses, 3 (1.3%) wound bleeding events, and 1 (0.4%) pressure sore. Nine (3.9%) patients received a secondary operation due to wound complications, including debridement in 5 patients, check bleeding in 2 patients, and flap reconstruction for pressure sore in one patient. The overall median hospital stay was 13 days (range 8–57 days), and the median wound healing time was 23 days (range 9–578 days). The median perineal wound healing time in the delayed healing group was 63.5 days (range 30–578 days).

**Table 2:** Operative outcome

	No. (%)	Range (SD)
Median operation time (mins)	240	115–590 (83.1)
Median blood loss (mL)	250	30–2300 (349.9)
Laparoscopic operation	28 (12.2)	
Resection of adjacent pelvic organs	32 (14.0)	
Perineal wound-related complication	91 (39.7)	
Delayed wound healing (>30 days)	86 (37.6)	
Superficial infection	27 (11.8)	
Dehiscence	22 (9.6)	
Deep or pelvic abscess	7 (3.1)	
Persistent discharging sinus	3 (1.3)	
Bleeding	3 (1.3)	
Pressure sore	1 (0.4)	
Re-operation for perineal wounds	9 (3.9)	
Median perineal wound healing time (days)	23	9–578 (65.1)
Median hospital stay (days)	13	8–57 (7.9)

We further analyzed risk factors for delayed perineal wound healing. Univariate analysis showed that patients with hypoalbuminemia had a statistically significant increased risk for delayed wound healing (10.5% *vs.* 26.7%, *P* = 0.001, Table 3). Preoperative neoadjuvant radiotherapy had a trend of increased risk for delayed perineal wound healing, although this difference was not statistically significant (28.7% vs. 40.7%, *P* = 0.061). Multivariable analysis showed that hypoalbuminemia (OR = 2.962, 95% CI = 1.437–6.102, *p* = 0.003) was an independent risk factor for delayed perineal wound healing.

**Table 3:** Univariate and multivariate analysis of risk factors for delayed healing of perineal wound

Variables	No delayed healing *n* = 143 (%)	Delayed healing *n* = 86 (%)	*P*	Multivariate	
				OR (95% CI)	*P*
Age > 65 (years old)	82 (57.3)	58 (67.4)	0.129		
Gender			0.170		
Male	96 (67.1)	50 (58.1)			
Female	47 (32.9)	36 (41.9)			
Hypertension	59 (41.3)	40 (46.5)	0.437		
Diabetic mellitus	21 (14.7)	18 (20.9)	0.223		
Congestive heart failure	24 (16.8)	14 (16.3)	0.921		
COPD	8 (5.6)	2 (2.3)	0.241		
Uremia	6 (4.2)	3 (3.5)	0.790		
Liver cirrhosis	3 (2.1)	0 (0.0)	0.176		
Smoking					
Previous	38 (26.6)	23 (26.7)	0.977		
Current	25 (17.5)	13 (15.1)	0.641		
BMI (kg/m^2^)					
Underweight (< 18.5)	5 (3.5)	4 (4.7)	0.595		
Overweight (> 25)	53 (37.1)	33 (38.4)	0.517		
Neoadjuvant radiotherapy	41 (28.7)	35 (40.7)	0.061	1.582 (0.889–2.816)	0.119
Hypoalbuminemia (< 3.5 g/dL)	15 (10.5)	23 (26.7)	0.001*	2.962 (1.437–6.102)	0.003*
Pre-op CEA > 5 ng/mL	47 (34.3)	32 (37.6)	0.613		
Resection of other pelvic organ	19 (13.3)	13 (15.1)	0.699		
Laparoscopic operation	14 (9.8)	14 (16.3)	0.147		
Operation time > 240 min	70 (49.0)	45 (52.3)	0.621		
Blood loss > 250 ml	62 (43.4)	41 (47.7)	0.525		

*COPD* chronic obstructive pulmonary disease, *BMI* body mass index, *CEA* carcinoembryonic antigen

*Statistically significant, *P* < 0.05

Risk factors for prolonged hospital stay were also analyzed, and patients with delayed wound healing had a significantly higher risk of prolonged hospital stay than those with normal wound healing (21.3% vs*.* 63.6%, *p* < 0.001, Table 4). The other risk factor for prolonged hospital stay included patients with more than one comorbidity (50.4% *vs.* 63.6%, *p* = 0.049). In the multivariable analysis, delayed wound healing (OR = 6.404, 95% CI = 3.508–11.694, *p* < 0.001) was an independent risk factor for prolonged hospital stay.

**Table 4:** Univariate and multivariate analysis of risk factors for prolonged hospital stay

Variables	Hospital stay ≤ 14 days *n* = 141 (%)	Hospital stay > 14 days *n* = 88 (%)	*P*	Multivariate	
				OR (95% CI)	*P*
Age > 65 (years old)	82 (58.2)	58 (65.9)	0.242		
Gender			0.976		
Male	90 (63.8)	56 (63.6)			
Female	51 (36.2)	32 (36.4)			
Comorbidity ≥ 1	71 (50.4)	56 (63.6)	0.049	1.720 (0.937–3.158)	0.080
Comorbidity ≥ 2	32 (22.7)	26 (29.5)	0.246		
Smoking					
Previous	35 (24.8)	26 (29.5)	0.432		
Current	23 (16.3)	15 (17.0)	0.885		
BMI (kg/m^2^)					
Underweight (< 18.5)	6 (4.3)	3 (3.4)	0.820		
Overweight (> 25)	51 (36.2)	35 (39.8)	0.464		
Neoadjuvant CCRT	52 (36.9)	24 (27.3)	0.133		
Hypoalbuminemia	19 (13.5)	19 (21.6)	0.108		
Pre-op CEA > 5 ng/mL	45 (33.3)	34 (39.1)	0.383		
Resection of other pelvic organ	18 (12.8)	14 (15.9)	0.505		
Laparoscopic operation	19 (13.5)	9 (10.2)	0.466		
Operation time > 240 min	70 (49.6)	45 (51.1)	0.826		
Blood loss > 250 ml	57 (40.4)	46 (52.3)	0.080	1.718 (0.942–3.134)	0.078
Delayed perineal wound healing	30 (21.3)	56 (63.6)	< 0.001*	6.404 (3.508–11.694)	< 0.001*

*CCRT* concurrent chemoradiotherapy, *BMI* body mass index, *CEA* carcinoembryonic antigen

## Discussion

Perineal wound complications are a long-lasting issue for APR patients. Removal of the rectum, anus, and sometimes nearby organs results in a large empty space, which is conducive to fluid accumulation and bacteria growth [7]. Moreover, the addition of preoperative radiotherapy may also cause tissue damage and reduce the blood supply [2, 17]. All of these reasons may interfere with wound healing and lead to delayed wound healing. Our study showed that the incidence of delayed perineal wound healing was 37.6%, and hypoalbuminemia was an independent risk factor for delayed perineal wound healing after APR. The consequence of delayed perineal wound healing was an increased risk of prolonged hospital stay. In other words, if we can reduce the risk of delayed perineal wound healing, the incidence of prolonged hospital stays will also decrease, and total medical costs will decrease after such an intervention.

Delayed perineal wound healing is thought to be associated with decreased quality of life, increased health care costs, and poor survival [18]. The median perineal wound healing time of all patients in our study was 23 days, which was shorter than that reported by Chessin et al. (39.5 days) [19]. The mean perineal wound healing time of all patients in our study was 46.38 days, which was comparable to that reported by Bullard et al. (3.8 weeks) [2] and Althumairi et al. (6.85 weeks) [4]. However, a marked diversity of healing times, which ranged from 9 to 578 days, was observed in our group. The median healing time in the delayed healing group was 63.5 days. Such a long healing time will not only prolong the hospital stay and increase medical costs but also delay any scheduled adjuvant treatment. Although flap reconstruction can be performed to minimize perineal wound complications and facilitate perineal wound healing, the additional procedure also increases the operation time and risk of complications at both the surgical and donor sites. In our study, hypoalbuminemia was an independent risk factor for delayed perineal wound healing (*p* = 0.003) based on multivariate analysis. The serum albumin level could represent a patient’s general nutritional status. Protein deficiency reflected by hypoalbuminemia contributes to reduced collagen formation and wound dehiscence and results in poor wound healing rate [20]. Low albumin has also been shown to contribute to poor wound healing in diabetic foot ulcers [21, 22]. An animal study demonstrated that the early administration of albumin could enhance wound healing in rats with burn injuries [23]. It is unknown whether the early administration of albumin will improve perineal wound healing after APR, but improving the patient’s nutritional status before operation will certainly benefit wound healing. Further clinical trials are needed to evaluate the efficacy of reducing the incidence of delayed perineal wound healing by correcting albumin levels before APR.

Previous studies suggested that radiotherapy was a major risk factor for delayed wound healing [2, 4, 12]. In our study, radiotherapy had a nonsignificant tendency for delayed wound healing. This may be related to the low percentage of patients receiving preoperative radiotherapy (33.2%) in our study. Patient factors were also crucial for wound healing [24]. Malnutrition, smoking, COPD, and obesity makes patients vulnerable to poor wound healing [18, 25]. Uremia is a common risk factor for poor wound healing [26], which might be related to impaired calcium-phosphorus metabolism and uremic toxins. Our results did not show relationships between comorbidities, BMI, and smoking and delayed perineal wound healing. The mean BMI was 24.03 kg/m^2^ in our patient group, which was lower than that (27.9 kg/m^2^) in a study by Hawkins et al. [18]. Only 6 (2.6%) patients had a BMI over 30 kg/m^2^. The case number in each subgroup in our study was limited, and more patients might be required to reach statistical significance.

The median hospital stay in our group was 13 days, which is also comparable to previous studies [4, 17, 27]. The risk factors of prolonged hospital stay in our study included the presence of comorbidities and the occurrence of delayed perineal wound healing. Delayed perineal wound healing was a strong predictive factor for prolonged hospital stay (*p* < 0.001). Thus, our efforts should be focused on reducing the incidence of delayed perineal wound healing. Vacuum-assisted closure was introduced for complex perineal wounds [28, 29]. When wound healing time is decreased, granulation tissue deposition is increased, which allows earlier wound closure, an earlier hospital discharge, and an earlier return to functional status. However, vacuum-assisted closure is not routinely used in our institute due to the lack of National Health Insurance support in Taiwan.

There are several limitations to our study. First, this was a single-institute analysis with some limited patient subgroup numbers, which may have resulted in nonsignificant differences in the risk factor analysis. Second, as this was a retrospective review, we were unable to accurately determine the true condition of the patients with perineal wound complications. The differences between the definitions of wound infection, wound dehiscence, and superficial or deep abscess formation are difficult to quantify. We chose the 30-day criterion because this was the time that the patient’s condition was checked by the attending surgeon prior to initiating postoperative adjuvant therapy. We believe that healing time is more objective for perineal wound complications in a retrospective analysis because it is difficult to diagnose infection, dehiscence, or sinus discharge simply from a chart review.

In conclusion, our data suggest that hypoalbuminemia (albumin < 3.5 g/dL) is an independent risk factor for delayed perineal wound healing. Moreover, delayed perineal wound healing is strongly related to prolonged hospital stay. Further studies for improving wound care are needed.

## Data Availability

The datasets used and/or analyzed during the current study are available from the corresponding author on reasonable request.

## Abbreviations

APR: Abdominoperineal resection
BMI: Body mass index
CEA: Carcinoembryonic antigen

## References

[1] WE. A method of performing abdomino-perineal excision for carcinoma of the rectum and of the terminal portion of the pelvic colon (1908). Lancet.2:1812-3. 10.3322/canjclin.21.6.361 5001853

[2] Bullard KM, Trudel JL, Baxter NN, Rothenberger DA (2005). Primary perineal wound closure after preoperative radiotherapy and abdominoperineal resection has a high incidence of wound failure. Dis Colon Rectum.

[3] Musters GD, Sloothaak DA, Roodbeen S, van Geloven AA, Bemelman WA, Tanis PJ (2014). Perineal wound healing after abdominoperineal resection for rectal cancer: a two-centre experience in the era of intensified oncological treatment. Int J Colorectal Disease.

[4] Althumairi AA, Canner JK, Gearhart SL, Safar B, Sacks J, Efron JE (2016). Predictors of perineal wound complications and prolonged time to perineal wound healing after abdominoperineal resection. World J Surg.

[5] Kokosis G, Sun Z, Avashia YJ, Adam MA, Levinson H, Erdmann D (2017). V-Y fasciocutaneous flap closure technique is a safe and efficacious alternative to primary closure of the perineal wound following abdominoperineal resection. Am J Surg.

[6] Shukla HS, Tewari M (2010). An evolution of clinical application of inferior pedicle based rectus abdominis myocutaneous flap for repair of perineal defects after radical surgery for cancer. J Surg Oncol..

[7] Holm T, Ljung A, Haggmark T, Jurell G, Lagergren J (2007). Extended abdominoperineal resection with gluteus maximus flap reconstruction of the pelvic floor for rectal cancer. Br J Surg.

[8] Christian CK, Kwaan MR, Betensky RA, Breen EM, Zinner MJ, Bleday R (2005). Risk factors for perineal wound complications following abdominoperineal resection. Dis Colon Rectum.

[9] Dinaux AM, Amri R, Berger DL (2017). Prone positioning reduces perineal infections when performing the miles procedure. Am J Surg.

[10] Persichetti P, Cogliandro A, Marangi GF, Simone P, Ripetti V, Vitelli CE (2007). Pelvic and perineal reconstruction following abdominoperineal resection: the role of gracilis flap. Ann Plast Surg.

[11] Miles W. E. (1971). A Method of Performing Abdomino-Perineal Excision for Carcinoma of the Rectum and of the Terminal Portion of the Pelvic Colon (1908). CA: A Cancer Journal for Clinicians.

[12] Spasojevic M, Mariathasan AB, Goscinski M, Thorgersen EB, Solbakken AM, Gullestad HP (2018). Vertical rectus abdominis musculocutaneous flap repair improves perineal wound healing after abdominoperineal resection for irradiated locally advanced rectal cancer. Ann Surg Oncol.

[13] Jones H, Moran B, Crane S, Hompes R, Cunningham C (2017). group L. The LOREC APE registry: operative technique, oncological outcome and perineal wound healing after abdominoperineal excision. Colorectal Dis.

[14] Artioukh DY, Smith RA, Gokul K (2007). Risk factors for impaired healing of the perineal wound after abdominoperineal resection of rectum for carcinoma. Colorectal Dis.

[15] Nissan A, Guillem JG, Paty PB, Douglas Wong W, Minsky B, Saltz L (2001). Abdominoperineal resection for rectal cancer at a specialty center. Dis Colon Rectum.

[16] Wang LW, Yang SH, Lin JK, Lin TC, Chan WK, Chen WS (2005). Pre-operative chemoradiotherapy with oral tegafur-uracil and leucovorin for rectal cancer. J Surg Oncol.

[17] Musters GD, Buskens CJ, Bemelman WA, Tanis PJ (2014). Perineal wound healing after abdominoperineal resection for rectal cancer: a systematic review and meta-analysis. Dis Colon Rectum.

[18] Hawkins AT, Berger DL, Shellito PC, Sylla P, Bordeianou L (2014). Wound dehiscence after abdominoperineal resection for low rectal cancer is associated with decreased survival. Dis Colon Rectum.

[19] Chessin DB, Hartley J, Cohen AM, Mazumdar M, Cordeiro P, Disa J (2005). Rectus flap reconstruction decreases perineal wound complications after pelvic chemoradiation and surgery: a cohort study. Ann Surg Oncol.

[20] Russell L (2001). The importance of patients' nutritional status in wound healing. Br J Nurs.

[21] Skrepnek GH, Armstrong DG, Mills JL (2014). Open bypass and endovascular procedures among diabetic foot ulcer cases in the United States from 2001 to 2010. J Vasc Surg.

[22] Shiraki T, Iida O, Takahara M, Soga Y, Yamauchi Y, Hirano K (2015). Predictors of delayed wound healing after endovascular therapy of isolated infrapopliteal lesions underlying critical limb ischemia in patients with high prevalence of diabetes mellitus and hemodialysis. Eur J Vasc Endovasc Surg.

[23] Kobayashi N, Nagai H, Yasuda Y, Kanazawa K (2004). The early influence of albumin administration on protein metabolism and wound healing in burned rats. Wound Repair Regen.

[24] Kamrava A, Mahmoud NN (2013). Prevention and management of nonhealing perineal wounds. Clin Colon Rectal Surg.

[25] El-Gazzaz G, Kiran RP, Lavery I (2009). Wound complications in rectal cancer patients undergoing primary closure of the perineal wound after abdominoperineal resection. Dis Colon Rectum.

[26] Maroz N, Simman R (2013). Wound healing in patients with impaired kidney function. J Am Coll Clin Wound Spec.

[27] Choudry U, Harris D (2013). Perineal wound complications, risk factors, and outcome after abdominoperineal resections. Ann Plast Surg.

[28] Schaffzin DM, Douglas JM, Stahl TJ, Smith LE (2004). Vacuum-assisted closure of complex perineal wounds. Dis Colon Rectum.

[29] Walma MS, Burbach JP, Verheijen PM, Pronk A, van Grevenstein WM (2016). Vacuum-assisted closure therapy for infected perineal wounds after abdominoperineal resection. A retrospective cohort study. Int J Surg.

